# Reply to Broomfield et al. Comment on “Jankovic et al. Understanding the Benefits of CO_2_ Laser Treatment for Vulvovaginal Atrophy. *Medicina* 2024, *60*, 1059”

**DOI:** 10.3390/medicina61050832

**Published:** 2025-04-30

**Authors:** Svetlana Jankovic, Marija Rovcanin, Ana Tomic, Aleksandar Jurisic, Zagorka Milovanovic, Milena Zamurovic

**Affiliations:** 1 Clinic for Gynecology and Obstetrics, Narodni Front, Kraljice Natalije 62, 11000 Belgrade, Serbia; 2 Faculty of Medicine, University of Belgrade, Dr Subotica Starijeg 8, 11000 Belgrade, Serbia; 3 Center for Radiology and Magnetic Resonance Imaging, University Clinical Center of Serbia, 11000 Belgrade, Serbia

Thank you for bringing these concerns [1] to our attention regarding our recent publication, “Understanding the benefits of CO_2_ laser treatment for vulvovaginal atrophy” published in the journal *Medicina* [2]. We appreciate the opportunity to address the points raised.

Firstly, regarding the similarity between our publication in *Medicina* [2] and our previous work in the journal *Healthcare* [3], we acknowledge that both studies address similar objectives and use similar outcome measures. However, it is important to note that while the focus of the *Healthcare* publication [3] included urinary and prolapse symptoms, the *Medicina* publication [2] specifically targeted vulvovaginal atrophy, providing a more detailed analysis of this condition; we aimed to explore vulvovaginal atrophy in greater detail, recognizing it as a significant issue affecting menopausal women. With that goal, we added a number of participants to the cohort of our *Healthcare* study [3], making it suitable for secondary analysis in our *Medicina*-published research [2]. Upon conducting the analysis and reviewing the results, we determined that the data could be presented separately, allowing us to highlight specific details that might not have received sufficient attention in a single publication. This decision was made to enhance the robustness of our findings and provide a more comprehensive analysis of the data mainly connected to VHIS, vaginal atrophy symptoms, and concordant sexual health.

We sincerely apologize for the oversight in not clearly stating that study published in the journal *Medicina* [2] involved a secondary analysis with an increased number of participants. Regrettably, we failed to properly note this in the methodology section of our publication, and we understand the potential impact this may have on the interpretation of our findings. We deeply regret any confusion or inconvenience this may have caused. We are fully committed to maintaining the highest standards of transparency and scientific rigor. To address this, we are available to provide any clarification if required and will cooperate fully with the journal to ensure that the matter is addressed appropriately. Additionally, we welcome any feedback or further steps required to ensure that the integrity of our work is upheld and that the scientific record remains accurate.

Concerning the conclusions drawn about the efficacy of CO_2_ laser therapy for VVA symptoms, we agree that the absence of a control group and randomization are limitations of our study. While our research aimed to explore the potential benefits of CO_2_ laser therapy, we acknowledge the importance of well-conducted randomized controlled trials (RCTs) and their role in establishing definitive conclusions. We appreciate the references to the existing RCTs and meta-analysis in the received comment and we will ensure to discuss these findings in greater detail in our future work.

In our case, the choice for conducting a pilot study rather than a more rigorous sham control study hinged on several critical factors. The primary reason for opting for a pilot study was the absence of prior research in the specific area in our country, hence why we thought it was prudent to first conduct a preliminary investigation. This approach allowed us to gather essential baseline data, identify potential challenges, and establish our study protocol for future control-based studies. Given that this study represents an initial exploration, it was necessary to ensure that the interventions could be safely and effectively administered. Conducting a pilot study helped us in excluding unforeseen risks or logistical issues that could compromise participant safety or data integrity in a full-scale sham control study. A pilot study provided us an opportunity to establish our study methodology, allowing for more precise data collection procedures (questionnaires) and outcome measures that were well received by our participants. Per our country’s economic status, implementing a sham control study without prior evidence can be resource intensive and potentially wasteful. Sham control studies typically require more participants and more complex logistics to ensure that blinding and control conditions are adequately maintained. Conducting a pilot study first allowed us to ensure that the full-scale study is both feasible and justified based on preliminary findings. Finally, ethical considerations played a significant role in the decision-making process. Conducting a sham control study involves subjecting participants to placebo interventions, which our ethics board upped to be ethically justified. Without prior research of the intervention’s potential efficacy and safety conduced on women in Serbia, it was not possible for us to justify the ethical balance of risks and benefits to our board of experts. A pilot study helped us to firmly establish preliminary evidence that can support the ethical rationale for a subsequent sham control study. In summary, the decision to conduct a pilot study rather than a sham control study was driven by the need to gather preliminary data in an uncharted research area in our country. Nonetheless, we acknowledge that a control study would be the ideal choice for rigorously testing the efficacy of the intervention now that the preliminary data have established its feasibility and safety.

We wish to address that the same remark and comment content was sent to both journals, with the *Medicina* journal receiving it first. After a few days, we received the exact same comment from the *Healthcare* journal, before we were even given the chance to respond to the *Medicina* journal editors within the allotted time (it was noted that we had time to respond until 6 October). This demonstrates not only impatience but also suggests malicious intent from external reviewers.

Furthermore, it is discriminatory to dismiss our research solely because it is not an RCT, particularly given the constraints of our country’s healthcare system and the conditions under which we operate. While RCTs are considered the gold standard, pilot studies are still valuable, as they provide essential preliminary data, help identify feasibility issues, and lay the groundwork for larger, more definitive trials. Pilot studies contribute meaningfully to scientific progress, especially in resource-limited settings, where conducting large-scale RCTs may not be immediately feasible.

Finally, our intent was not to overlook the broader context of existing research but to contribute additional insights into the potential benefits of CO_2_ laser therapy. We value the constructive feedback, and we are committed to advancing this area of research with rigorous methodologies and transparent reporting. Once again, we deeply regret any confusion or inconvenience the above-noted reasons may have caused. We hope this response addresses the concerns raised and reaffirms our commitment to scientific integrity and advancing knowledge in this field. Thank you once again for your valuable feedback.
